# Cross-genera SSR transferability in cacti revealed by a case study using *Cereus* (Cereeae, Cactaceae)

**DOI:** 10.1590/1678-4685-GMB-2017-0293

**Published:** 2019-02-21

**Authors:** Juliana Rodrigues Bombonato, Isabel Aparecida Silva Bonatelli, Gislaine Angélica Rodrigues Silva, Evandro Marsola Moraes, Daniela Cristina Zappi, Nigel P. Taylor, Fernando Faria Franco

**Affiliations:** 1 Universidade Federal de São Carlos Universidade Federal de São Carlos Departamento de Biologia SorocabaSP Brazil Departamento de Biologia, Centro de Ciências Humanas e Biológicas, Universidade Federal de São Carlos, Sorocaba, SP, Brazil; 2 Universidade de São Paulo Universidade de São Paulo Faculdade de Filosofia, Ciências e Letras de Ribeirão Preto Programa de Pós-graduação em Biologia Comparada Ribeirão PretoSP Brazil Programa de Pós-graduação em Biologia Comparada, Faculdade de Filosofia, Ciências e Letras de Ribeirão Preto, Universidade de São Paulo, Ribeirão Preto, SP, Brazil; 3 Instituto Tecnológico Vale Instituto Tecnológico Vale Museu Paraense Emilio Goeldi BelémPA Brazil Instituto Tecnológico Vale, Museu Paraense Emilio Goeldi, Coord. Botânica, Belém, PA, Brazil; 4 Singapore Botanic Gardens (National Parks Board) Singapore Botanic Gardens (National Parks Board) Singapore Republic of Singapore Singapore Botanic Gardens (National Parks Board), Singapore, Republic of Singapore

**Keywords:** Cactaceae, Cereus, cross-genera, SSR markers, Transferability

## Abstract

The study of transferability of simple sequence repeats (SSR) among closely related species is a well-known strategy in population genetics, however transferability among distinct genera is less common. We tested cross-genera SSR amplification in the family Cactaceae using a total of 20 heterologous primers previously developed for the genera *Ariocarpus, Echinocactus, Polaskia* and *Pilosocereus,* in four taxa of the genus *Cereus*: *C. fernambucensis* subsp. *fernambucensis*, *C. fernambucensis* subsp. *sericifer*, *C. jamacaru* and *C. insularis*. Nine microsatellite loci were amplified in *Cereus* resulting in 35.2% of success in transferability, which is higher than the average rate of 10% reported in the literature for cross-genera transferability in eudicots. The genetic variation in the transferred markers was sufficient to perform standard clustering analysis, indicating each population as a cohesive genetic cluster. Overall, the amount of genetic variation found indicates that the transferred SSR markers might be useful in large-scale population studies within the genus *Cereus*.

## Introduction

Simple sequence repeats (SSR) or microsatellites are, in general, non-coding regions commonly found in Eukaryote genomes composed of tandemly arranged repeat motifs from 1 to 6 base pairs (Oliveira *et al.*, 2006). SSRs are useful molecular markers for several applications in population genetics and breeding studies, as they frequently exhibit high levels of polymorphism, in addition to their abundance and random distribution across and throughout genomes. In plants, SSR loci have been used for several purposes, for example, estimates of genetic diversity (Zhu *et al.*, 2016), intra- and interspecific gene flow (Palma-Silva *et al.*, 2011; Pinheiro *et al.*, 2014), biogeographical distributions (Beatty and Provan 2011), phylogenetic relationships (Mehmood *et al.*, 2016), genetic mapping (Tan *et al.*, 2016), and conservation (Gómez-Fernández *et al.*, 2016).

An alternative to overcome time consuming and costly development of a new set of SSR primers for a target species is to carry out the transferability of SSR primers among related species (Barbará *et al.*, 2007; Lavor *et al.*, 2013; Nogueira *et al.*, 2015). The rate of success in this approach (i.e., heterologous amplification) depends on the nucleotide similarity among the flanking regions of different species. Therefore, it is expected that there will be a higher rate in heterologous amplification among taxa with recent divergence times. In plants, this technique has been widely adopted for a great variety of eudicots (e.g., Haerinasab *et al.*, 2016; Mengistu *et al.*, 2016), where the average rate of success at infrageneric level is around 60% (Barbará *et al.*, 2007). The rate of cross-genera transferability is around 10% in eudicots (Barbará *et al.*, 2007), but the levels of success may reach values above 50% in some plants (Satya *et al.*, 2016).

Taking into account the recent divergence within Cactaceae, as well as its emergence as an informative model to study diversification in xeric habitats (Arakaki *et al.*, 2011), the aim of this study was to perform cross-genera SSR amplification in four closely related taxa of the genus *Cereus* (Cactaceae; Cereeae) occurring in eastern Brazil: *C. fernambucensis* subsp. *fernambucensis*, *C. fernambucensis* subsp. *sericifer*, *C. insularis* and *C. jamacaru.* A previous phylogenetic analysis based on plastid DNA placed *C. jamacaru* as a member of a polytomic clade, sister of the monophyletic clade composed by *C. fernambucensis* and *C. insularis* (Franco *et al.*, 2017a). In this study, we selected a set of 11 SSR loci originally described for *Ariocarpus bravoanus* (Hughes *et al.*, 2008), *Echinocactus grusonii* (Hardesty *et al.*, 2008) and *Polaskia chichipe* (Otero-Arnaiz *et al.*, 2004) that were recently transferred to *Cereus* species cultivated in different urban areas, including *C. hildmannianus* (Martin 2011; Fernandes *et al.*, 2016). An additional nine SSR loci described for *Pilosocereus machrisii* (Perez *et al.*, 2011) were included in this investigation.

We sampled 122 individuals from representative populations of *C. jamacaru*, *C. insularis* and *C. fernambucensis* (Table 1), besides one individual of *C. hildmannianus* (Salto, SP; 23.99, 47.33; SORO 2746) as a positive control in the initial tests. Genomic DNA was extracted from the radicular tissue using the Qiagen DNeasy Plant Mini Kit (Qiagen, Hilden, Germany). As the samples from localities S82 and S83 are geographically (~34 km) and genetically close, sharing the same unique alleles and comprising a cohesive genetic group, we decided to join the individuals from the two populations in a single sample, hereinafter referred to as S82/S83.

**Table 1:** Geographical localities of the populations from three species of *Cereus* used in this work.

Species	Voucher	Geographic Coordinates (S, W)	N
*C. fernambucensis* subsp. *fernambucensis* Lem.			
Arraial do Cabo, RJ (S80) - Southern group^*^	SORO 2663	-22.97, -42.03	19
Maracajaú, RN (S104) - Northern group^*^	SORO 4529	-5.39, -35.31	20
Una, BA (S114) - Northern group^*^	SORO 2675	-15.11, -39.00	20
*C. fernambucensis* subsp. *sericifer* Ritt.			
Santa Maria Madalena, RJ (S82) - Southern group^*^	SORO 2665	-21.95, -42.03	12
Itaoacara, RJ (S83) - Southern group^*^	SORO 2666	-21.65, -42.09	4
Águia Branca, ES (S88) - Northern group^*^	SORO 2749	-19.06, -40.69	10
*C. jamacaru* DC.			
Conceição de Feira, BA (S113)	HUEFS 33711	-12.59, -38.99	18
*C. insularis* Hmsl.			
Fernando de Noronha, PE (S115D)	SORO 2677	-3.85, -32.40	19

* The classification in Southern and Northern group is based on phylogeographic data available for *C. fernambucensis* (Franco *et al.*, 2017b) / N = number of individuals per populations.

Initial amplification tests were performed using a subsample of 12 individuals, with slight modifications on PCR conditions as described by Albert and Schmitz (2002), Don *et al.* (1991), and Perez *et al.* (2011). The reactions were performed in 10 μL of total PCR volume including 0.5 U of *Taq* DNA Polymerase (Promega), 1X *Taq* Buffer (5X Colorless GoTaq® Flexi Buffer), 0.2 μM dNTPs, and primer and MgCl_2_ concentrations varying when necessary. We considered a locus successfully transferred when the PCR products were clearly visualized in 3% agarose gels and showed a product size compatible with the range described for that locus. The loci successfully amplified were then genotyped in the total sample (Table 1) using PAGE (denaturing polyacrylamide gel) with concentrations varying between 6% to 9%, according to expected allele size. To visualize the alleles, the gels were stained with silver nitrate. The percentage of transferability success was estimated according to the number of individuals amplified in each locus.

The occurrence of null alleles, allele drop-out, and stutter bands was evaluated with Micro-Checker 2.2.3 software (Van Oosterhout *et al.*, 2004). The number of alleles per locus (*N*_a_), effective number of alleles (*n*_e_), expected (*H*_e_) and observed (*H*_o_) heterozygosities, private alleles, and percentage of polymorphic loci were estimated using GenAlEx 6.5 software (Peakall and Smouse, 2012). The inbreeding coefficient (*F*_IS_) per population was calculated using FSTAT 2.9.3.2 (Goudet, 1995), assuming α = 0.01 and α = 0.001 (Lavor *et al.*, 2013; Ribeiro *et al.*, 2014). Deviations from Hardy-Weinberg equilibrium (HWE) and linkage disequilibrium (LD) were investigated using the Arlequin 3.5.1.3 program (Excoffier and Lischer, 2010). We used the sequential Bonferroni correction for multiple testing with α = 0.05 (Rice, 1989) to minimize statistical errors. Genetic differentiation among populations was quantified by *F*_ST_ (Weir and Cockerham, 1984) estimated in FSTAT 2. 9. 3. 2 (Goudet, 1995) and corrected for null alleles in FreeNA (Chapuis and Estoup, 2007). The pairwise chord distances (Dc, Cavalli-Sforza and Edwards, 1967) between populations was estimated in FreeNA software (Chapuis and Estoup, 2007), and the resulting matrix was then used as input to generate a Neighbor-Joining dendrogram (NJ) (Saitou and Nei, 1987) in Populations 1.2.32 software (Langella, 1999). To explore genetic structure in our data we performed: a Principal Coordinate Analysis (PCoA) in GenAlEx 6.5 (Peakall and Smouse, 2012); a global and a hierarchical Analysis of Molecular Variance (AMOVA) in Arlequin 3.5.1.3 (Excoffier and Lischer, 2010); and a Bayesian clustering analysis in STRUCTURE 2.3.4 (Pritchard *et al.*, 2000). The latter was implemented using 10 simultaneous and independent runs with 10^6^ generations of MCMC (25% as burn-in). The K-values tested ranged from 1 to 8. To find the best K we used ΔK statistics (Evanno *et al.*, 2005) in Structure Harvester. The results of the independent runs for the best K were combined in Clumpp (Jakobsson and Rosenberg, 2007), and were graphically displayed with Distruct (Rosenberg, 2004).

From the 20 tested loci (Table S1), nine (*mAbR 28* from *A. bravoanus*; *mEgR 02*, *mEgR 76* and *mEgR 78* from *E. grusonii* and *Pmac82*, *Pmac84*, *Pmac108*, *Pmac146* and *Pmac149* from *P. machrisii*) showed positive results in transferability for at least one species (Table S2), resulting in 35.16% of success in transferability. Except for *mEgR 02*, the allele size for all loci was congruent with the expected size (Table S2). We were not able to amplify five SSR loci previously transferred to *Cereus* (*Pchi 21*, *Pchi 47*, *Pchi 54*, *mAbR 42* and *mAbR 77*) (Martin, 2011; Fernandes *et al.*, 2016), even after several attempts to modify PCR conditions (Table S3). This result is likely related to nucleotide differences among the flanking regions of the samples used in this work, preventing primer annealing.

The percentage of polymorphic loci ranged from 44.4% (populations S113 and S82/S83) to 77.8% (location S80) (Table 2). In contrast with the expectation of reduced levels of genetic diversity at transferred SSR loci (e.g. Goldstein and Pollock, 1997; Jan *et al.*, 2012; Moodley *et al.*, 2015), we found higher levels of polymorphism for some loci (*Pmac82* in all populations*, mEgR 78* in S88 and *mEgR 02* in S80) than those reported in the original description (Table 2). No locus showed significant heterozygosity deficiency in relation to expectations of HWE after Bonferroni correction. Inbreeding coefficient estimates (*F*_IS_) provided no significant result (Table 2). The locus *Pmac108* showed high levels of observed heterozygosity in all populations, excepting S82/S83 (Table 2). Private alleles were found in populations S113, S88, S115D, S114, S104 and S82/83 (Table S4). The LD analysis results between polymorphic loci were not statistically significant after Bonferroni correction.

**Table 2:** Genetic diversity indices: Number of samples (N), Number of alleles (*Na*), Effective allele number (*Ne*), Observed heterozygosity (*Ho*), Expected heterozygosity (*He*), absence (-) and presence (+) of null alleles, and *F*_*IS*_ values per loci and population are shown.

Locus	N	*Na*	*Ne*	*Ho*	*He*	Null Allele	*F*_*IS*_	*p*-value
Population S113							0.06	0.26
*Pmac82*	18	3	1.57	0.33	0.36	-		
*Pmac108*	13	5	2.38	0.62	0.58	-		
*Pmac149*	18	5	1.81	0.50	0.45	-		
*mEgR 02*	16	1	1.00	0.00	0.00	-		
*mAbR 28*	16	1	1.00	0.00	0.00	-		
*mEgR 76*	18	2	1.12	0.00	0.11	-		
Média	16.5	2.83	1.48	0.24	0.25			
Population S80							0.08	0.13
*Pmac82*	19	2	1.17	0.16	0.15	-		
*Pmac84*	18	3	2.66	0.78	0.62	-		
*Pmac108*	18	5	2.61	0.67	0.62	-		
*Pmac146*	17	3	2.34	0.41	0.57	-		
*mEgR 02*	16	3	1.68	0.25	0.41	-		
*mAbR 28*	14	4	1.57	0.43	0.36	-		
*mEgR 76*	17	1	1.00	0.00	0.00	-		
*mEgR 78*	17	2	1.12	0.00	0.11	-		
Média	17.0	2.56	1.77	0.34	0.36			
Population S82/S83							0.05	0.35
*Pmac82*	16	2	1.44	0.38	0.30	-		
*Pmac84*	15	1	1.00	0.00	0.00	-		
*Pmac108*	13	1	1.00	0.00	0.00	-		
*Pmac146*	12	5	1.71	0.17	0.42	++		
*mEgR 02*	14	1	1.00	0.00	0.00	-		
*mAbR 28*	11	1	1.00	0.00	0.00	-		
*mEgR 76*	14	2	1.51	0.00	0.34	++		
*mEgR 78*	16	2	2.00	1.00	0.50	-		
Média	13.88	1.88	1.33	0.19	0.19			
Population S88							0.24	0.01
*Pmac82*	10	5	3.13	0.40	0.68	++		
*Pmac84*	10	2	1.60	0.10	0.38	-		
*Pmac108*	8	6	4.74	0.50	0.79	++		
*Pmac146*	9	4	2.00	0.56	0.50	-		
*mEgR 02*	7	1	1.00	0.00	0.00	-		
*mAbR 28*	5	1	1.00	0.00	0.00	-		
*mEgR 76*	10	1	1.00	0.00	0.00	-		
*mEgR 78*	9	4	3.00	0.89	0.67	-		
Média	8.50	3.00	2.18	0.31	0.38	-		
Population S115D							0.59	1.00
*Pmac82*	19	2	1.05	0.05	0.05	-		
*Pmac84*	19	2	1.70	0.58	0.41	-		
*Pmac108*	19	2	1.98	0.89	0.49	-		
*Pmac146*	18	6	3.27	1.00	0.69	-		
*mEgR 02*	18	2	1.74	0.61	0.42	-		
*mAbR 28*	15	1	1.00	0.00	0.00	-		
*mEgR 76*	17	1	1.00	0.00	0.00	-		
*mEgR 78*	18	2	2.00	1.00	0.50	-		
Média	17.88	2.25	1.72	0.52	0.32	-		
Population S104							0.18	0.99
*Pmac82*	20	4	1.60	0.45	0.37	-		
*Pmac84*	20	2	1.72	0.60	0.42	-		
*Pmac108*	20	6	3.15	1.00	0.68	-		
*Pmac146*	18	5	2.19	0.61	0.54	-		
*mEgR 02*	20	1	1.00	0.00	0.00	-		
*mAbR 28*	20	2	1.66	0.25	0.40	-		
*mEgR 76*	20	1	1.00	0.00	0.00	-		
*mEgR 78*	19	1	1.00	0.00	0.00	-		
Média	19.63	2.75	1.67	0.36	0.30	-		
Population S114							0.01	0.52
*Pmac82*	20	2	1.05	0.05	0.05	-		
*Pmac84*	20	4	2.02	0.55	0.50	-		
*Pmac108*	19	4	2.06	0.68	0.52	-		
*Pmac146*	13	2	1.83	0.54	0.45	-		
*mEgR 02*	20	1	1.00	0.00	0.00	-		
*mAbR 28*	20	1	1.00	0.00	0.00	-		
*mEgR 76*	20	1	1.00	0.00	0.00	-		
*mEgR 78*	15	2	1.92	0.27	0.48	-		
Média	18.38	2.13	1.48	0.26	0.25			

*p* < 0.01 / 0.001 = significant, and *p* > 0.01 / 0.001 = not significant.

**Figure 1:**
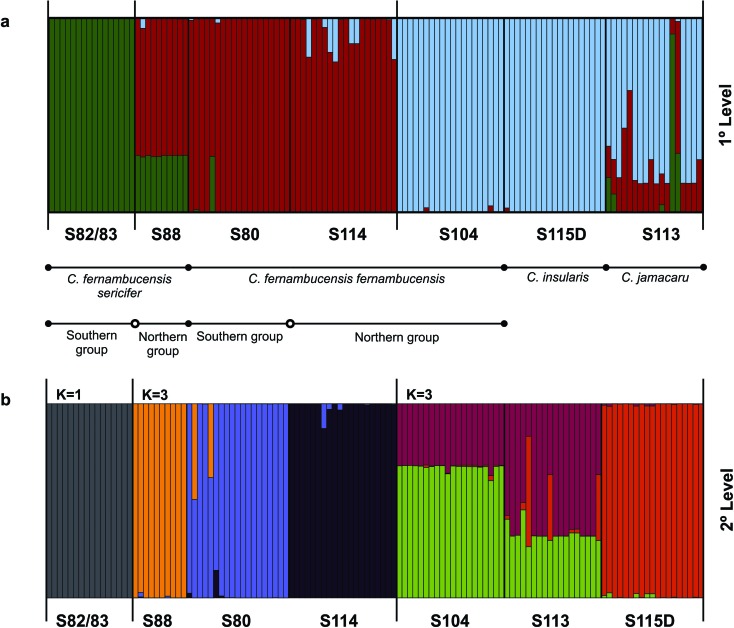
Population differentiation in STRUCTURE, (a) results for K = 3 on the first level structure, and (b) separating each population as a distinct genetic group. The southern and northern population groups of C*. fernambucensis* subsp. *sericifer* and *C. fernambucensis* subsp. *fernambucensis* are based on phylogeographic circumscription (Franco *et al.*, 2017b).

The FreeNA-corrected estimate of global *F*_ST_ was 0.44, ranging from 0.12 (*Pmac82*) to 0.79 (*mEgR 02*) (Table S5). Clustering analyses (NJ, PCoA, STRUCTURE) have shown somewhat distinct results (Figure 1 and Figure S1). However, the results from AMOVA suggest that the three clusters recovered by STRUCTURE better explain the genetic variation structuring in our data (Table 3), as follows: 1) S113, S014 and S115D populations; 2) S80, S88 and S114 populations; 3) S82/S83 (Figure 1a). To investigate sub-structuration within our data, we performed a STRUCTURE analysis for each cluster, which resulted in each location being a cohesive genetic group (Figure 1b). Although we have not done an extensive geographic sampling for each studied taxon, and the number of markers is relatively low, some clustering results recovered here agree with previous phylogeographic hypotheses established for *C. fernambucensis* and *C. insularis* based on cpDNA and the *PHYC* gene (Franco *et al.*, 2017b). The close relationship of *C. jamacaru* and *C. fernambucensis* subsp. *fernambucensis* (S104) deserves additional investigation, but seems to be a spurious grouping as a result of the reduced number of sampled populations.

**Table 3:** Global and hierarchical Analysis of Molecular Variance (AMOVA). For hierarchical AMOVA the a priori groups are based on taxonomic circumscription, NJ phenogram, PCoA and STRUCTURE.

Groups	Fixation Indexes	Source of Variation	Percentege of Variation
Global AMOVA			
All populations only one group	Φ_ST_ = 0.34460	Among populations within group	34.46
Taxonomic circumscription			
S113, S115D, S82/S83 with S88, S80 with S104 and S114	Φ_CT_ = 0.01385	Among groups	1.38
	Φ_SC_ = 0.33731	Among populations within groups	33.26
NJ Phenogram			
S113, S80 with S82/83 and S88, S104 with S115D, S114	Φ_CT_ = -0.25331	Among groups	-25.33
	Φ_SC_ = 0.45546	Among populations within groups	57.08
PCoA			
	Φ_CT_ = -0.26648	Among groups	-26.65
S113, all others populations (S80, S82/83, S88, S104, S114 and S115D)	Φ_SC_ = 0.38531	Among populations within groups	48.80
STRUCTURE			
S82/S83, S80 with S88 and S114, S104 with S113 and S115D	Φ_CT_ = 0.34737	Among groups	34.74
	Φ_SC_ = 0.09764	Among populations within groups	6.37

The estimated success in transferability observed in this study (35.16%) was higher than the average of 10% found in cross-genera transferability studies published between 1997 and mid-2006 (see Barbará *et al.*, 2007). However, this is not an uncommon result, as similar findings or even higher levels of cross-genera transferability were observed in some groups of plants (Satya *et al.*, 2016). In the family Iridaceae, for example, a success of 77% was observed in cross-amplification between genera (Miz *et al.*, 2016). In the family Malvaceae cross-genera SSR transferability varied from 71% to 92% (Satya *et al.*, 2016), while in Euphorbiaceae these percentages ranged from 9.5% to 59.1% (Whankaew *et al.*, 2011). Evidently, genera are taxonomic categories mainly based on morphological instead of genetic information, and different levels of phylogenetic divergence must be embedded within each genus. Therefore, the success in cross-genera transferability may vary highly depending on the target organism. On the other hand, it is expected that the success in cross-amplification should be a function of phylogenetic distance, at least regarding genetic differentiation (Barbará *et al.*, 2007).

This expectation was not clearly observed here considering cactus phylogeny (Hernández-Hernández *et al.*, 2014). We observed similar success in heterologous amplification using primers described for relatively distantly (*A. bravoanus* and *E. grusonii* – four transferred of nine tested) or closely related species (*P. machrisii* – five transferred of 11 tested). It is worth highlighting that for those loci from *A. bravoanus*, *E. grusonii,* and *P. chichipe* we had a previous expectation of positive cross-amplification, as they were formerly transferred for some *Cereus* species (Martin, 2011; Fernandes *et al.*, 2016). Nevertheless, higher levels of cross-genera amplification in the cactus family might be a widespread tendency, as the main lineage divergences and species radiation events within this family are thought to have occurred in the last 10 Myr (Arakaki *et al.*, 2011; Hernández-Hernández *et al.*, 2014; Silva *et al.*, 2018). Even with remarkable morphological distinctness among cactus species, resulting in more than 120 recognized genera (Hunt *et al.*, 2006), these recent divergence times increase the possibility of heterologous amplification in the family Cactaceae due to the expected similarities in the flanking SSR regions among different species. Evidently, this is not a rule, as even here we found discordance between some results obtained by Martin (2011) and Fernandes *et al.* (2016), which were likely due to nucleotide differences in flanking regions of the distinct samples. At any rate, this information should be taken into consideration to encourage cross-genera transferability studies in Cactaceae, which, despite their potential, are still relatively scarce in this family (Table S6).

The genus *Cereus* constitutes an interesting biological model to perform evolutionary studies, and efforts were employed to screen informative molecular markers in this genus (Silva *et al.*, 2016; Romeiro-Brito *et al.*, 2016) to solve species level phylogeny (Franco *et al.*, 2017a) and to investigate population differentiation and phylogeography (Franco *et al.*, 2017b; Silva *et al.*, 2018). Our results are in line with these endeavors, supplying additional molecular markers that can be useful for estimating genetic diversity and gene flow in target *Cereus* species. Furthermore, considering the rate of success in transferability, our results should encourage cactus researchers interested in using the increasing number of SSR loci that have been described for representatives of this highly diverse and relatively overlooked plant family (e.g., Bonatelli *et al.*, 2015; Fava *et al.*, 2016).

## Supplementary material

The following online material is available for this article:

Figure S1 Neighbor-joining phenogram.

Table S1 Characteristics of 20 microsatellite loci tested for transferability.

Table S2 Transferability results of the SSR markers for populations.

Table S3 PCR conditions for the SSR markers transferred.

Table S4 Private alleles (> 10% frequency) found in each locus per population.

Table S5 F-statistics by locus for all populations and FST corrected by ENA method.

Table S6 Results of a literature survey.
