# Supplementary material for: Cross-genera SSR transferability in cacti revealed by a case study using Cereus (Cereeae, Cactaceae)
Source: Genet Mol Biol. 2019 Feb 21;42(1):87–94. doi: 10.1590/1678-4685-GMB-2017-0293 (PMC6428128; doi:10.1590/1678-4685-GMB-2017-0293)
Supplement: Supplementary file 1 [file 1415-4757-GMB-1678-4685-GMB-2017-0293-20190123-suppl2.pdf]

Supplementary Material to "Cross-genera SSR transferability in cacti revealed by a case study using *Cereus* (Cereeae, Cactaceae)"

Table S1 - Characteristics of 20 microsatellite loci tested for transferability in *Cereus* species.

Source of Species Description	Locus	Repeat motif	Primer Sequence (5' – 3')	Reference	PCR Program	Annealing Temperature (°C)
Polaskia chichipe	*Pchi 21*	$(AG)_{10}(AAG)_2C(CA)_2$	F: CGTTTAGCCCCTCTTTCTCC<br>R: GTTCCCAACTGACCGACAAC	Otero-Arnaiz *et al.*, 2004	Touchdown (Don *et al.*, 1991)	65°C
	Pchi 47	$(TC)_{10}$	F: GTCCTTGTGGCTAGCCCTTT<br>R: CCATTTCTCTCGCCATCTG	Otero-Arnaiz *et al.*, 2004	Albert and Schmitz, 2002	53°C
	Pchi 54	$(AG)_4CG(AG)_{11}$	F: CCTTGAGCTTTGACATTGAGA<br>R: GGAAGGTTTTCATTGGATGAG	Otero-Arnaiz *et al.*, 2004	Albert and Schmitz, 2002	52°C
Ariocarpus bravoanus	*mAbR 28*	$(CT)_5(AT)_3(GT)_8GA(GT)_5$	F: CCATAAGCTGTGGTGGGTCT<br>R: ATTTTAAAGCTCCCCCTCCA	Hughes *et al.*, 2008	Albert and Schmitz, 2002	52°C
	mAbR 42	$(TG)_{15}$	F: GGGCAATTCACTATGCACAA<br>R: TTGTCCCACCTTCCCTATTG	Hughes *et al.*, 2008	Albert and Schmitz, 2002	52°C
	mAbR 77	$(CA)_5CG(CA)_5TG(CA)_{22}(TA)_3$	F: CGGGGAAGGAATAATCCAAG<br>R: ATGTGCCGTTTGCAATCTTT	Hughes *et al.*, 2008	Albert and Schmitz, 2002	52°C
Echinocactus grusonii	*mEgR 02*	$(AG)_7$	F: TGGGTTGGAGAAGTGGAAG<br>R: CGGTGTGAGGCTTCATTTG	Hardesty *et al.*, 2008	Albert and Schmitz, 2002	52°C
	mEgR 76	$(AG)_{10}AC(AG)_2AC(AG)_7$	F: TCACAATTTGGAAGGAAGCA<br>R: GTGAGCAAAGGGCTGATTTC	Hardesty *et al.*, 2008	Touchdown (Don *et al.*,, 1991)	65°C
	mEgR 78	$(AG)_{13}GAG(CA)_3$	F: AGCCCAAAGCCCAACTTATT<br>R: TGCATGCAATCATAAGGTTTTC	Hardesty *et al.*, 2008	Albert and Schmitz, 2002	52°C
Pilosocereus machrisii	*Pmac82*	$(GAG)_5(GAA)_2(GAG)_2GAA(GAG)_2$	F: GTAAAAGAGGAGGATGGAGAGG<br>R: CTTCTTCTTGCTAGGTTTCTCG	Perez *et al.*, 2011	Perez *et al.*, 2011	56°C
	Pmac84	$(AG)_9CG(AG)_2$	F: CATAAATTGCAGAAATGAGGAC<br>R: AGGTAAACCGCTAACTCGATG	Perez *et al.*, 2011	Perez *et al.*, 2011	60°C
	Pmac85	$(AG)_6AC(AG)_3AC(AG)_4$	F: CCCCTCACTTCTCCAATCTC<br>R: TCCCTCCTTCTTTTGTGTTC	Perez *et al.*, 2011	Perez *et al.*, 2011	58°C
	Pmac101	$(TC)_{15}TATG(TA)_3$	F: TGATAGCTGCAACGATGTC<br>R: CATTTGTTTTGTTGTTTGCTTCAC	Perez *et al.*, 2011	Perez *et al.*, 2011	49°C
	Pmac102	$(AG)_9$	F: TCTATAAGTGCCGATGGATGC<br>R: CACACCTCACTCCCAACCTC	Perez *et al.*, 2011	Perez *et al.*, 2011	59°C
	Pmac108	$(AG)_{14}(TG)_7$	F: TGAATGTCTGCCATTAGAAAGC<br>R: TAATCGCCCCAAAGAAGAAC	Perez *et al.*, 2011	Perez *et al.*, 2011	50°C
	Pmac128	$(TC)_5TT(TC)_{10}(AC)_{10}$	F: GTGTTGATTGTACTCTTCAG<br>R: CTAACCCTTTGTATACATGC	Perez *et al.*, 2011	Perez *et al.*, 2011	59°C
	Pmac130	$(AG)_7CA(AG)_{12}$	F: GAGGTGCCAATAAATCG<br>R: TGTCACGCAATCTTGAACC	Perez *et al.*, 2011	Perez *et al.*, 2011	58°C
	Pmac135	$(TC)_5TG(TC)_{12}$	F: ACCAGAATGAGCTCAGCTGTAG<br>R: CCTAGCTAGCAGAATCAGAGTGAAGAC	Perez *et al.*, 2011	Perez *et al.*, 2011	60°C
	Pmac146	$(AG)_{20}$	F: ACCCGACATCCCACTTGTAG<br>R: TAGTCTGAAACGGAGCAAGG	Perez *et al.*, 2011	Perez *et al.*, 2011	54°C
	Pmac149	$(TC)_{19}$	F: TTCATCCTGCTTTTGAAGTTTG<br>R: TGATGGATTAGGATTGACCTG	Perez *et al.*, 2011	Perez *et al.*, 2011	59°C
