# Supplementary material for: Cross-genera SSR transferability in cacti revealed by a case study using Cereus (Cereeae, Cactaceae)
Source: Genet Mol Biol. 2019 Feb 21;42(1):87–94. doi: 10.1590/1678-4685-GMB-2017-0293 (PMC6428128; doi:10.1590/1678-4685-GMB-2017-0293)
Supplement: Supplementary file 2 [file 1415-4757-GMB-1678-4685-GMB-2017-0293-20190123-suppl3.pdf]

Supplementary Material to "Cross-genera SSR transferability in cacti revealed by a case study using *Cereus* (Cereeae, Cactaceae)"

Table S2 - Transferability results of the SSR markers for populations S113 (*C. jamacaru*); S115D (*C. insularis*); S80, S104 and S114 (*C. fernambucensis* subsp. *fernambucensis*), S82/S83 and S88 (*C. fernambucensis* subsp. *Sericifer*). The negative (-) and the positive (+) signs mean failure and success in transferability, respectively. The reference source of each locus can be observed in Table S1.

	Populations							Allelic Size Range (bp)	
**SSR Locus**	**S113**	**S115D**	**S80**	**S104**	**S114**	**S82/S83**	**S88**	**Expected**	**Observed**
Pchi 21	-	-	-	-	-	-	-	124	-
Pchi 47	-	-	-	-	-	-	-	120	-
Pchi 54	-	-	-	-	-	-	-	170	-
mAbR 28	+	+	+	+	+	+	+	207-222*	166-186
mAbR 42	-	-	-	-	-	-	-	196–217	-
mAbR 77	-	-	-	-	-	-	-	346-374	-
mEgR 02	+	+	+	+	+	+	+	260-280**	155-178
mEgR 76	+	+	+	+	+	+	+	376-396	360-366
mEgR 78	-	+	+	+	+	+	+	148-242***	128-252
Pmac82	+	+	+	+	+	+	+	81-93	81-98
Pmac84	-	+	+	+	+	+	+	80-84	76-84
Pmac85	-	-	-	-	-	-	-	90 – 98	-
Pmac101	-	-	-	-	-	-	-	108-114	-
Pmac102	-	-	-	-	-	-	-	188 – 120	-
Pmac108	+	+	+	+	+	+	+	109-135	102-130
Pmac128	-	-	-	-	-	-	-	138-154	-
Pmac130	-	-	-	-	-	-	-	141 – 149	-
Pmac135	-	-	-	-	-	-	-	126-140	-
Pmac146	-	+	+	+	+	+	+	113-155	82-136
Pmac149	+	-	-	-	-	-	-	142-154	132-140

*According to Fernandes *et al.* (2016) the expected size was 103-176.

** According to Fernandes *et al.* (2016) the size was < 100.

*** According to Fernandes *et al.* (2016) the size range was 250-288.
