# Supplementary material for: Cross-genera SSR transferability in cacti revealed by a case study using Cereus (Cereeae, Cactaceae)
Source: Genet Mol Biol. 2019 Feb 21;42(1):87–94. doi: 10.1590/1678-4685-GMB-2017-0293 (PMC6428128; doi:10.1590/1678-4685-GMB-2017-0293)
Supplement: Supplementary file 3 [file 1415-4757-GMB-1678-4685-GMB-2017-0293-20190123-suppl4.pdf]

Supplementary Material to "Cross-genera SSR transferability in cacti revealed by a case study using *Cereus* (Cereeae, Cactaceae)"

Table S3 - PCR conditions for the SSR markers transferred with respective alleles size per population. The negative sign (-) means failure in transferability.

**Species**	**Populations**	**Conditions**	***mAbR 28***	***mEgR 02***	***mEgR 76***	***mEgR 78***	***Pmac82***	***Pmac84***	***Pmac108***	***Pmac146***	***Pmac149***
C. fernambucencis subsp. *fernambucensis*	S80	T (°C)	52	55	65-55 (TD)	52	56	52	49	54	-
		$MgCl_2$ (µM)	3.0	3.0	2.5	2.5	1.5	1.5	1.5	1.5	-
		Primer (µM)	0.3	0.5	1.0	0.5	0.25	0.25	0.25	0.25	0.25
		Size (bp)	176-186	155-168	360	232-236	84-90	78-82	104-130	128-136	-
	S104	T (°C)	52	52	65-55 (TD)	52	56	60	50	54	-
		$MgCl_2$ (µM)	3.0	3.0	2.5	2.5	1.5	1.5	1.5	1.5	-
		Primer (µM)	0.3	0.5	1.0	0.5	0.25	0.25	0.25	0.25	0.25
		Size (bp)	166-176	166	360	250	87-98	78-80	104-118	82-130	-
	S114	T (°C)	52	52	65-55 (TD)	52	56	60	50	54	-
		$MgCl_2$ (µM)	3.0	3.0	2.5	2.5	1.5	1.5	1.5	1.5	-
		Primer (µM)	0.3	0.5	1.0	0.5	0.25	0.25	0.25	0.25	0.25
		Size (bp)	176	166	360	236-250	87-98	78-84	104-110	116-130	-
C. fernambucencis subsp.*sericifer*	S82/S83	T (°C)	52	52	65-55 (TD)	52	54	53	49	53	-
		$MgCl_2$ (µM)	3.0	3.0	2.5	2.5	1.5	1.5	1.5	1.5	-
		Primer (uM)	0.3	0.5	1.0	0.5	0.25	0.25	0.25	0.25	0.25
		Size (bp)	176	178	360-366	236-252	81	78	126	126-134	-
	S88	T (°C)	52	52	65-55 (TD)	52	56	53	50	53	-
		$MgCl_2$ (µM)	3.0	3.0	2.5	2.5	1.5	1.5	1.5	1.5	-
		Primer (µM)	0.3	0.5	1.0	0.5	0.25	0.25	0.25	0.25	0.25
		Size (bp)	176	168	360	128-254	87-90	76-78	102-126	128-136	-
C. jamacaru	S113	T (°C)	52	52	65-55 (TD)	-	56	-	47/49	-	59
		$MgCl_2$ (µM)	3.0	3.0	2.5	-	1.5	-	1.5	-	1.5
		Primer (µM)	0.3	0.5	1.0	0.5	0.25	0.25	0.25	0.25	0.25
		Size (bp)	176	166	360-366	-	81-84	-	120-130	-	132-140
C. insularis	S115D	T (°C)	52	52	65-55 (TD)	52	56	60	50	54	-
		$MgCl_2$ (µM)	3.0	3.0	2.5	2.5	1.5	1.5	1.5	1.5	-
		Primer (µM)	0.3	0.5	1.0	0.5	0.25	0.25	0.25	0.25	0.25
		Size (bp)	176	166-172	360	236-250	87-98	78-80	106-110	110-126	-
