# Supplementary material for: Cross-genera SSR transferability in cacti revealed by a case study using Cereus (Cereeae, Cactaceae)
Source: Genet Mol Biol. 2019 Feb 21;42(1):87–94. doi: 10.1590/1678-4685-GMB-2017-0293 (PMC6428128; doi:10.1590/1678-4685-GMB-2017-0293)
Supplement: Supplementary file 4 [file 1415-4757-GMB-1678-4685-GMB-2017-0293-20190123-suppl5.pdf]

Supplementary Material to "Cross-genera SSR transferability in cacti revealed by a case study using *Cereus* (Cereeae, Cactaceae)"

Table S4 - Private alleles (>10% frequency) found in each loci per population.

Population	Loci	Allele	Frequency
S113	*Pmac108*	124	0.15
	Pmac149	138	0.17
	Pmac149	140	0.72
	mEgR 02	156	0.12
	mAbR 28	186	0,11
S82/83	*mEgR 02*	178	1.00
	mEgR 78	252	0.50
S88	*Pmac82*	82	0.20
	Pmac82	85	0.10
	Pmac84	76	0.25
	Pmac108	102	0.25
	Pmac146	132	0.22
	mEgR 78	128	0.11
	mEgR 78	238	0.33
	mEgR 78	254	0.11
S115D	*mEgR 02*	172	0.31
S104	*Pmac108*	116	0.10
	mAbR 28	166	0.27
S114	*Pmac84*	84	0.12
	Pmac146	116	0.35
