# Supplementary material for: Cross-genera SSR transferability in cacti revealed by a case study using Cereus (Cereeae, Cactaceae)
Source: Genet Mol Biol. 2019 Feb 21;42(1):87–94. doi: 10.1590/1678-4685-GMB-2017-0293 (PMC6428128; doi:10.1590/1678-4685-GMB-2017-0293)
Supplement: Supplementary file 5 [file 1415-4757-GMB-1678-4685-GMB-2017-0293-20190123-suppl6.pdf]

Supplementary Material to "Cross-genera SSR transferability in cacti revealed by a case study using *Cereus* (Cereeae, Cactaceae)"

Table S5 - F-statistics by locus for all populations and FST corrected by ENA method (Chapuis and Estoup, 2007).

SSR Locus	F_{IT}	F_{IS}	F_{ST}	F_{ST} Corrected
Pmac82	0.13	0.04	0.09	0.12
Pmac84	0.35	-0,16	0.43	0.43
Pmac108	0.23	-0,25	0.38	0.38
Pmac146	0.36	-0,02	0.37	0.35
mEgR 02	0.78	-0,03	0.79	0.79
mEgR 76	1,00	1,00	0.71	0.64
mEgR 78	0.29	-0,41	0.49	0.46
mAbR 28	0.33	0.19	0.17	0.19

F_{ST} global = 0.44 / F_{ST} corrected (95% confidence interval (CI) 0.33 to 0.57)
