# Supplementary material for: Cross-genera SSR transferability in cacti revealed by a case study using Cereus (Cereeae, Cactaceae)
Source: Genet Mol Biol. 2019 Feb 21;42(1):87–94. doi: 10.1590/1678-4685-GMB-2017-0293 (PMC6428128; doi:10.1590/1678-4685-GMB-2017-0293)
Supplement: Supplementary file 6 [file 1415-4757-GMB-1678-4685-GMB-2017-0293-20190123-suppl1.pdf]

Supplementary Material to "Cross-genera SSR transferability in cacti revealed by a case study using *Cereus* (Cereeae, Cactaceae)"

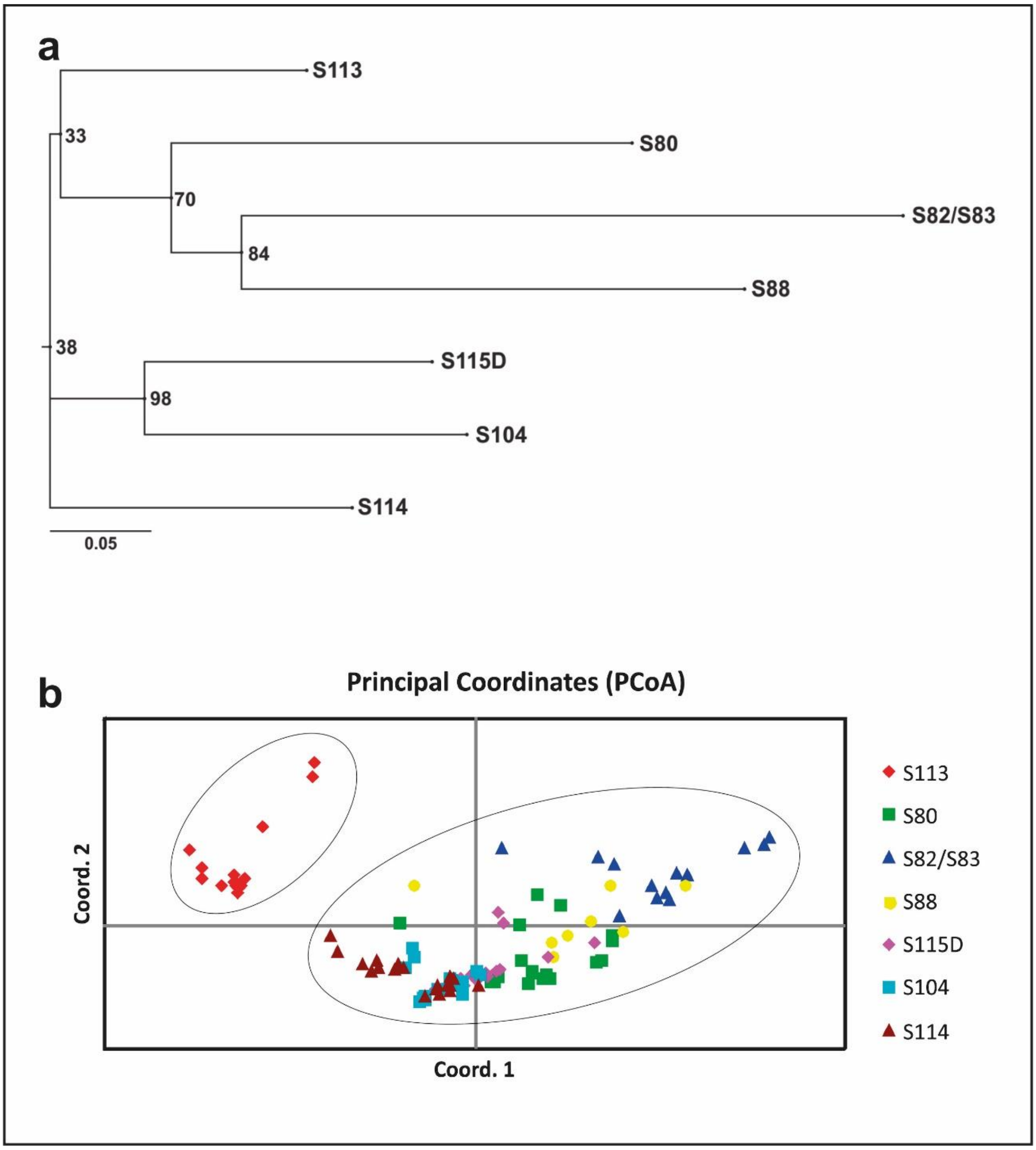

Figure S1 - Neighbor-joining phenogram, (a) showing the relationship (genetic distances) among populations based on the transferred SSR markers. Branch supports (bootstrap) are presented near the nodes. The scale bar represents genetic distances calculated according to Dc "chord-distance" (Cavalli-Sforza and Edwards, 1967); (b) shows the distribution of individuals sampled against the first two axes of PCoA, where Coord 1 and Coord 2 explains, respectively, 26.52% and 21.91% of total genetic variation.
