# Supplementary material for: Cross-genera SSR transferability in cacti revealed by a case study using Cereus (Cereeae, Cactaceae)
Source: Genet Mol Biol. 2019 Feb 21;42(1):87–94. doi: 10.1590/1678-4685-GMB-2017-0293 (PMC6428128; doi:10.1590/1678-4685-GMB-2017-0293)
Supplement: Supplementary file 7 [file 1415-4757-GMB-1678-4685-GMB-2017-0293-20190123-suppl7.pdf]

Supplementary Material to "Cross-genera SSR transferability in cacti revealed by a case study using *Cereus* (Cereeae, Cactaceae)"

Table S6 - Results of a literature survey (up to April/2017) for articles on Cactaceae SSR transferability in ISI Web of Science.

Keywords*	**Type**	**N° of article**	**Authors**
Microsatellite transferability	Topic	855	-
Microsatellite cross-amplification	Topic	727	-
Cactaceae microsatellite transferability	Topic	2	Monteiro *et al.*, (2015) Biochem Syst Ecol 58:7-12<br>Fernandes *et al.*, (2016) Biochem Syst Ecol 64:38-45
Cactaceae microsatellite cross-amplification	Topic	7	Otero-Arnaiz *et al.*, (2004) Mol Ecol Notes 4:265-267<br>Solorzano *et al.*, (2009) Mol Ecol Res 9:156–158<br>Perez *et al.*, (2011) Am J Bot 98:204-206.<br>Moraes *et al.*, (2012) Genetica 140:277–285<br>Moraes *et al.*, (2014) Genet Mol Res 13: 10359-10366<br>Bonatelli *et al.*, (2015) PLoSONE 23:3044–3063<br>Fava *et al.*, (2016) Biochem Syst Ecol 66:19-23

*Note: A similar search was performed using the SSR term rather than microsatellite and the same results were retrieved.
